# Health Tract, No. 187

**Published:** 1864-02

**Authors:** 


HEALTH TRACT, No. 187.

### CORRECTING CHILDREN.

NOT long ago an editor in the northern part of the State of New-York, told his son, about eleven years old, that he would whip him in the course of a few hours, and locked him in an upper room until he had leisure to do so. When the boy heard the father coming, he became so alarmed that he jumped out of the window and broke his neck.

About a year ago a mother punished her little daughter, of eight years, by shutting her up in a dark closet; the child became so frightened that convulsions were induced, which resulted in death. In another case of a similar character, the result was still more calamitous, for the child became epileptic, and so remained for a long life afterward.

The object of parental correction should be the ultimate good of the child; and to make it effective,

1. The character of the punishment should be according to the disposition and temperament of the child.

2. The punishment should be in proportion to the nature of the offense.

3. The punishment should be inflicted with the utmost self-possession; for if done in a towering passion it takes the character of revenge; the child sees it and resists it with defiance, stubbornness, or with a feeling of being the injured or oppressed party.

4. Punishment should never be threatened, for one of two results, both unfortunate, are certain: the promise will not be kept and the child loses confidence in parental assertions; or the child's mind, dwelling upon what is expected, suffers a lengthened torture, imagination always aggravating the severity of the chastisement, and the child gradually learns to startle at every event which is at all likely to usher in the correction, and the foundation is laid for that fearfulness of the future which is the bane of all human happiness; and in some cases the severity of the expected suffering looms up so largely under the influence of a distempered imagination, that, as in the case of the editor's child, suicide is considered the lesser evil. It is nothing less than a savage barbarity for any parent to hold the mind of a child in a state of terrorism for a single hour, let alone for days and weeks.

5. Never correct a child by scolding, admonition, or castigation in the presence of any other person whatever. It is an attack on its self-esteem which provokes resistance and passion. Let grown persons recollect how ill they bear even deserved reproof in the presence of others.

6. Never punish a child twice for any one offense; it is a great injustice, a relic of barbarism, and always either discourages or hardens. Make each settlement final in itself, and don't be forever harping on what is past.

7. Punishment should not be inflicted in any case without placing clearly before the child's mind the nature of the aggravation, and that the sole design of the chastisement or reproof is his present and future welfare.

8. In all cases where punishment is decided upon, it should be prompt, or deferred, according to the degree of aggravation or palpable wrong. It is almost always better to defer; but in such cases threaten nothing, say nothing, do nothing which indicates in the slightest degree that any thing is to come. And when the time does come, do not alarm the child with any show of preparation, but gradually and affectionately bring up the whole matter; place it in its true, just, and clear light, and act accordingly; and always, as much as possible, appeal to the child's conscience, to its sense of right, to its magnaminity, to its benevolence toward men, and its gratitude toward God.

